# Associations Between Social Appearance Anxiety, Disordered Eating Attitudes, and Orthorexia Nervosa: A Cross‐Sectional Study

**DOI:** 10.1002/brb3.71690

**Published:** 2026-08-17

**Authors:** Zeynep Uzdil, Seda Kaya, Gökmen Zararsız, Funda Pınar Çakıroğlu

**Affiliations:** ^1^ Faculty of Health Sciences Department of Nutrition and Dietetics Ondokuz Mayıs University Samsun Türkiye; ^2^ Faculty of Health Sciences Department of Nutrition and Dietetics Tokat Gaziosmanpaşa University Tokat Türkiye; ^3^ Faculty of Medicine Department of Biostatistics Erciyes University Kayseri Türkiye; ^4^ Faculty of Health Sciences Department of Nutrition and Dietetics Ankara University Ankara Türkiye

**Keywords:** body image, eating disorders, disordered eating attitudes, orthorexia nervosa, social appearance anxiety

## Abstract

**Objective:**

Disordered eating attitudes and orthorexia nervosa (ON) represent important public health concerns. Social appearance anxiety may constitute a key psychological factor underlying these patterns. This study aimed to examine social appearance anxiety as associated with disordered eating attitudes and ON in a large adult sample.

**Method:**

This cross‐sectional study was conducted using an online questionnaire. A total of 827 adults (614 female [74.2%] and 213 male [25.8%]) were included in this study, with a mean age of 30.1 ± 7.6 years. Data were collected using the Eating Attitudes Test‐26 (EAT‐26), the Orthorexia Nervosa Inventory (ONI), and the Social Appearance Anxiety Scale (SAAS). Group comparisons were performed using independent samples t‐tests and one‐way analysis of variance, and linear regression analyses were conducted to assess the predictive role of social appearance anxiety.

**Results:**

Social appearance anxiety scores were significantly higher among females, single individuals, and participants with lower physical activity levels (*p* < 0.05). Participants at risk for disordered eating and those with higher ON tendencies exhibited significantly elevated SAAS scores (*p* < 0.01). Linear regression analyses indicated that social appearance anxiety was independently associated with both disordered eating attitudes and orthorexia nervosa after adjustment for sociodemographic variables (*p* < 0.001).

**Discussion:**

Social appearance anxiety appears to be a significant psychological factor associated with both disordered eating attitudes and ON. These findings underscore the importance of addressing appearance‐related concerns in the prevention and management of maladaptive eating behaviors.

## Graphical abstract

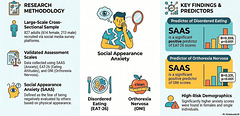

## 1 Introduction

Disordered eating attitudes are considered a significant public health problem, particularly due to their increasing prevalence among adolescents and young adults. The rise of sociocultural pressures and idealized body standards is associated with the development of maladaptive eating behaviors in individuals (Dane and Bhatia 2023; Holland and Tiggemann 2016; Ladwig et al. 2026). These attitudes encompass a range of unhealthy eating‐related thoughts and behaviors, including restrictive dieting, skipping meals, and compulsive overeating, which may not meet the diagnostic criteria for eating disorders but constitute an important risk for their development (Fitzsimmons‐Craft et al. 2016). The etiology of disordered eating attitudes is multifactorial, involving biological, psychological, and sociocultural factors (Girard et al. 2018; Stice and Shaw 2002). However, the rise of “clean eating” and healthy lifestyle trends in recent years has led to an excessive and rigid focus on consuming healthy and high‐quality foods in some individuals, paving the way for the emergence of the concept of orthorexia nervosa (ON) (Bratman 2017).

Orthorexia nervosa is a maladaptive eating behaviors pattern characterized by an excessive preoccupation with healthy and “clean” eating and has attracted increasing attention in recent years (Bratman 2017; Cena et al. 2019). Individuals exhibiting ON tendencies focus on food quality rather than quantity, develop strict dietary rules, and avoid foods they perceive as unhealthy. This can lead to negative consequences such as decreased quality of life, social isolation, and physical health problems (Donini et al. 2004; Douma et al. 2021; McGovern et al. 2021).

Although ON shares certain characteristics with eating disorders—such as perfectionism, body‐related concerns, and difficulties in emotion regulation—it is not currently recognized as a distinct diagnostic category in the Diagnostic and Statistical Manual of Mental Disorders‐5 (DSM‐5) or International Classification of Diseases‐11 (ICD‐11) (American Psychiatric Association 2013; WHO 2018). Therefore, the nature of the relationship between ON and disordered eating attitudes remains controversial, and it is not yet clear whether ON is an independent construct or a precursor or variant of eating disorders (Dunn and Bratman 2016; Strahler et al. 2018).

Recent studies have shown significant associations between various types of anxiety and both disordered eating behaviors and ON (Hussenoeder et al. 2021; Yılmaz and Dundar 2022). In this context, social appearance anxiety is defined as the fear of being negatively evaluated by others based on one's physical appearance and stands out as an important psychological factor associated with body dissatisfaction and maladaptive eating behaviors (Hart et al. 2008; Levinson and Rodebaugh 2012). Social appearance anxiety is not limited solely to body weight and shape but refers to a broader construct encompassing concerns about an individual's general physical characteristics (Argon 2014). The internalization of ideal body images presented by the media and social environment can increase appearance‐focused anxiety in individuals, paving the way for disordered eating habits (Aparicio‐Martinez et al. 2019; Grabe et al.^2008^; Merino et al. 2024).

Previous studies have independently reported associations among social appearance anxiety, disordered eating attitudes, and orthorexia nervosa (Eriksson et al. 2008; Hayes et al. 2017; Kiss‐Leizer et al. 2019). Moreover, orthorexic tendencies have consistently been associated with disordered eating attitudes across different populations (Brytek‐Matera et al. 2020; Farchakh et al. 2019; Haddad et al. 2019; Parra‐Fernández et al. 2018). However, studies simultaneously examining these constructs within a single analytical framework remain scarce. Furthermore, few studies have investigated whether social appearance anxiety constitutes a common psychological factor underlying both disordered eating attitudes and orthorexia nervosa while accounting for relevant sociodemographic characteristics. Therefore, the present study aimed to examine the role of social appearance anxiety in disordered eating attitudes and orthorexia nervosa in a large adult sample using the Orthorexia Nervosa Inventory (ONI), a multidimensional instrument that comprehensively assesses the behavioral, emotional, and impairment‐related dimensions of orthorexia nervosa. Based on previous evidence, we hypothesized that social appearance anxiety would be positively associated with both disordered eating attitudes and orthorexia nervosa, including their respective subscales, and would independently predict both outcomes after adjustment for relevant sociodemographic characteristics.

## 2 Materials and Methods

### 2.1 Study Design and Participants

This cross‐sectional study was conducted using an online questionnaire. The required sample size was calculated using the G*Power software (Version 3.1) with a 95% confidence level and a 5% margin of error, resulting in a minimum sample size of 820 participants (Turel et al. 2018). Participants were informed about the purpose of the study, and informed consent was obtained electronically. Data confidentiality and voluntary participation were ensured. The inclusion criteria were as follows: (1) being 18 years of age or older; (2) not being pregnant or breastfeeding; and (3) without clinically diagnosed eating disorders, chronic diseases, or psychiatric disorders.

The study was conducted in accordance with the principles of the Declaration of Helsinki and was approved by the Clinical Research Ethics Committee of Ondokuz Mayıs University (Approval Number: B.30.2.ODM.0.20.08/117‐89).

### 2.2 Data Collection

Data were collected using an online questionnaire created via Google Forms. The questionnaire, in the Turkish language, was shared with participants living in Türkiye. Prior to the main data collection, the survey was pilot‐tested with 10 participants who were not included in the final sample to estimate the time required to complete the questionnaire (approximately 10–15 min) and to identify potentially unreliable responses (e.g., excessively fast or slow completion times).

Participants were recruited using a convenience sampling method through social media platforms, including WhatsApp, Instagram, Telegram, and Facebook. The survey link was disseminated via these platforms, and participants were encouraged to share it with their networks. The recruitment materials included information about the purpose of the study, the procedures, and the estimated completion time, along with a web link that provided access to the survey after participants provided online informed consent. Participants were informed of their right to decline participation or withdraw at any time without consequences. Researchers’ contact information was provided for any questions or concerns related to the study.

The questionnaire included items related to sociodemographic characteristics (including gender, marital status, and education level); lifestyle characteristics (including physical activity level, and smoking status); chronic disease status; self‐reported body weight and height; the Social Appearance Anxiety Scale (SAAS); the Eating Attitudes Test‐26 (EAT‐26); and the Orthorexia Nervosa Inventory (ONI). Physical activity level was categorized into four groups: (1) no or very little activity; (2) mild (walking 1–3 days per week); (3) moderate (brisk walking ≥3 days per week); and (4) very active (intense exercise ≥6 days per week) (Kaya et al. 2024a). Body weight and height were self‐reported by participants, and body mass index (BMI) was calculated as weight (kg) divided by height squared (m^2^). BMI was classified according to the World Health Organization criteria for adults (WHO 2004).

### 2.3 Social Appearance Anxiety Scale

Social appearance anxiety was measured using the SAAS, developed by Hart et al. (2008). The Turkish validity and reliability study of the scale was conducted by Doğan (2010). The SAAS consists of 16 items rated on a 5‐point Likert scale, with total scores ranging from 16 to 80. The total score ranges from 16 to 80, with higher scores indicating greater social appearance anxiety. There is no universally accepted clinical cut‐off score for the SAAS; therefore, the total score was analyzed as a continuous variable in the present study. The Cronbach's alpha was 0.96 in this study.

### 2.4 Eating Attitudes Test‐26

Disordered eating attitudes were assessed using the 26‐item Eating Attitudes Test (EAT‐26), originally developed by Garner and Garfinkel (1979) and later adapted into its short form (Garner et al. 1982). The Turkish validity and reliability of the EAT‐26 have been established by Ergüney‐Okumuş and Sertel‐Berk (2019). The EAT‐26 is a 6‐point Likert‐type scale designed as a screening tool to identify individuals at risk for disordered eating attitudes rather than to diagnose clinical eating disorders. The test consists of three subscales: “dieting”, “bulimia and food preoccupation”, and “oral control”. The total score ranges from 0 to 78, and a score of ≥20 indicates a higher risk of disordered eating attitudes. In the present study, Cronbach's alpha was 0.81.

### 2.5 Orthorexia Nervosa Inventory

The tendency of ON was evaluated with the ONI which was developed by Oberle et al. (2021) in this study (Oberle et al. 2021). The Turkish validated versions of the ONI were conducted by Kaya et al. (2022). There are four consensus diagnostic criteria for determining ON (Criterion A, B, C, and D), and some scales related with ON not measure Criterion C (Kaya et al., 2022). Because it is a new scale that evaluates ON with all diagnostic criteria, including Criterion C—physical disorders. The scale consists of 24 items and is a 4‐point Likert‐type scale. As no universally accepted diagnostic cut‐off score has been established for the ONI, participants were categorized into low‐ and high‐orthorexia nervosa tendency groups according to the sample‐specific median ONI score for comparative analyses. This categorization was used solely for statistical comparisons and does not represent a clinical classification. The Cronbach's alpha was 0.91 in this study.

### 2.6 Statistical Analysis

Continuous variables were expressed as mean ± standard deviation (SD), and categorical variables were presented as number (*n*) and percentage (%). The normality of the data was evaluated using skewness and kurtosis coefficients (−2 to +2) (George and Mallery 2000). Differences in SAAS scores according to two independent variables were analyzed using the independent samples *t*‐test, and Cohen's formula *d* was used to determine effect size. For SAAS scores based on more than two independent variables, a one‐way analysis of variance (ANOVA) test and to determine effect size eta‐squared (*η* ^2^) were used. Conducted following a significant ANOVA test, Tukey's HSD test was used when the assumption of homogeneity of variances was met, and Tamhane's T2 test was used when the assumption of homogeneity of variances was not met. Linear regression analyses were performed to examine the associations between social appearance anxiety, disordered eating attitudes, and orthorexia nervosa tendency. Two adjusted models were constructed: Model 1 was adjusted for age and gender, and Model 2 was adjusted for marital status and physical activity level. Statistical analyses were conducted using SPSS version 26.0, and figures were generated using R software. A *p*‐value of <0.05 was considered statistically significant.

## 3 Results

This cross‐sectional study included 827 adults aged 18–64 years (mean age of 30.1 ± 7.6), comprising 614 females (74.2%) and 213 males (25.8%), all of whom completed the online survey. Table 1 presents the distribution of the SAAS scores according to participants' sociodemographic and lifestyle characteristics. The mean SAAS score was significantly higher among females compared to males (33.9 ± 15.1 vs. 30.9 ± 13.9, *p* = 0.013). Single participants exhibited significantly higher SAAS scores than married participants (35.2 ± 15.8 vs. 29.9 ± 12.7, *p* < 0.001). A statistically significant difference in SAAS scores was observed across education levels (*p* < 0.001). Participants with high school and undergraduate education reported higher SAAS scores compared with those holding postgraduate degrees. No significant differences in SAAS scores were found according to the presence of chronic disease (*p* = 0.494), body mass index categories (*p* = 0.222), or smoking status (*p* = 0.510). SAAS scores differed significantly according to physical activity level (*p* = 0.014). Participants reporting no or very little physical activity had higher SAAS scores than those engaging in mild physical activity.

**TABLE 1:** Comparison of SAAS Scores across sociodemographic and lifestyle characteristics.

Variables	n (%)	Mean ± SD	*t*/F	Cohen's *d/η^2^*	*p*
Gender			2.486	0.203	0.013*
Female	614 (74.2)	33.9 ± 15.1			
Male	213 (25.8)	30.9 ± 13.9			
Marital status			5.310	0.362	< 0.001*
Single	501 (60.6)	35.2 ± 15.8			
Married	326(39.4)	29.9 ± 12.7			
Education level			6.206	0.022	< 0.001*
Primary school	12 (1.5)	(30.4 ± 12.9)^ab^			
High school	61 (7.4)	(36.9 ± 15.5)^a^			
Undergraduate	245 (29.6)	(35.7 ± 15.3)^a^			
Postgraduate	509 (61.5)	(31.5±14.4)^b^			
Chronic diseases			−0.684	−0.074	0.494
No	726 (87.8)	32.9 ± 14.8			
Yes	101 (12.2)	34.0 ± 15.5			
BMI			1.467	0.005	0.222
Underweight	42 (5.1)	30.8 ± 12.6			
Normal	550 (66.5)	32.6 ± 14.2			
Overweight	183 (22.1)	34.3 ± 16.7			
Obesity	528 (6.3)	35.9 ± 16.5			
Physical activity			3.553	0.013	0.014^*^
No or very little	266 (32.2)	(35.5 ± 15.9)^a^			
Mild	393 (47.5)	(31.7 ± 13.7)^b^			
Moderate	152 (18.4)	(32.9 ± 15.8)^ab^			
Very active	16 (1.9)	(31.2 ± 11.3)^ab^			
Smoking			−0.659	−0.067	0.510
Non‐user	697 (84.3)	32.9 ± 14.6			
User	130 (15.7)	33.9 ± 16.3			

a‐b: There is no difference between groups with the same letter on the same rows. *p < 0.05.

Abbreviations: BMI, body mass index; SAAS, Social Appearance Anxiety Scale.

The distribution of SAAS scores according to eating attitudes and ON tendency is presented in Table 2. Participants classified as being at risk for disordered eating based on EAT‐26 scores had significantly higher SAAS scores than those with normal eating attitudes (41.4 ± 17.9 vs. 31.9 ± 14.0, *p* < 0.001). Similarly, participants with ONI scores above the median value (37 points), representing a high tendency toward orthorexia nervosa, had significantly higher SAAS scores than those with ONI scores at or below the median (Table 2).

**TABLE 2:** Comparison of SAAS Scores Across Eating Attitude and ON tendency.

Variables	*n* (%)	SAAS	*P*
Eating attitude			< 0.001^*^
Normal	721 (87.2)	31.9 ± 14.0	
Risk	106 (12.8)	41.4 ± 17.9	
ON tendency			0.004^*^
Low	406 (49.1)	31.6 ± 13.8	
High	421 (50.9)	34.6 ± 15.7	

*p < 0.05.

Abbreviations: EAT‐26, Eating Attitudes Test‐26; ON, Orthorexia Nervosa; SAAS, Social Appearance Anxiety Scale.

The relationships between social appearance anxiety, eating attitudes, and orthorexia nervosa were further examined using linear regression analyses (Table 3). In unadjusted models, SAAS was a significant positive associated factor of both orthorexia nervosa inventory (ONI) scores (B = 0.281, *p* < 0.001) and EAT‐26 scores (B = 0.366, *p* < 0.001). These associations remained statistically significant after adjustment for age and gender (Model 1) and further adjustment for age, gender, marital status, and physical activity level (Model 2). In the fully adjusted model, SAAS continued to predict ONI scores (B = 0.331, *p* < 0.001) and EAT‐26 scores (B = 0.359, *p* < 0.001).

**TABLE 3:** Linear regression analysis between SAAS with eating attitude and ON tendency.

Dependent variable	Model	Coefficient	B	SB	*t*	*p*	95% Confidence Interval		$R^{2}$	Adj‐$R^{2}$
							Lower Bound	Upper Bound		
ONI	Unadjusted model	(Constant)	22.085		10.721	**< 0.001**	18.041	26.128	0.036	0.034
		SAAS	0.281	0.189	5.523	**< 0.001**	0.181	0.381		
	Adjusted model^1^	(Constant)	30.840		10.593	**< 0.001**	25.126	36.555	0.085	0.082
		SAAS	0.322	0.216	6.430	**< 0.001**	0.224	0.420		
	Adjusted model^2^	(Constant)	34.569		10.972	**< 0.001**	28.385	40.753	0.099	0.094
		SAAS	0.331	0.222	6.633	**< 0.001**	0.233	0.429		
EAT‐26	Unadjusted model	(Constant)	29.391		37.357	**< 0.001**	27.847	30.935	0.044	0.043
		SAAS	0.366	0.210	6.177	**< 0.001**	0.250	0.483		
	Adjusted model^1^	(Constant)	42.086		17.113	**< 0.001**	37.258	46.913	0.079	0.076
		SAAS	0.351	0.201	6.014	**< 0.001**	0.236	0.465		
	Adjusted model^2^	(Constant)	41.081		14.932	**< 0.001**	35.681	46.481	0.093	0.087
		SAAS	0.359	0.206	6.157	**< 0.001**	0.245	0.474		

^1^Adjusted based on age, gender, ^2^Adjusted based on age, gender, marital status, physical activity. Adj‐$R^{2}$: Adjusted determination coefficient. Abbreviations: B, beta coefficient; EAT‐26, Eating Attitudes Test‐26; ONI, Orthorexia Nervosa Inventory; SAAS, Social Appearance Anxiety Scale; SB, standardized beta coefficient

**FIGURE 1:**
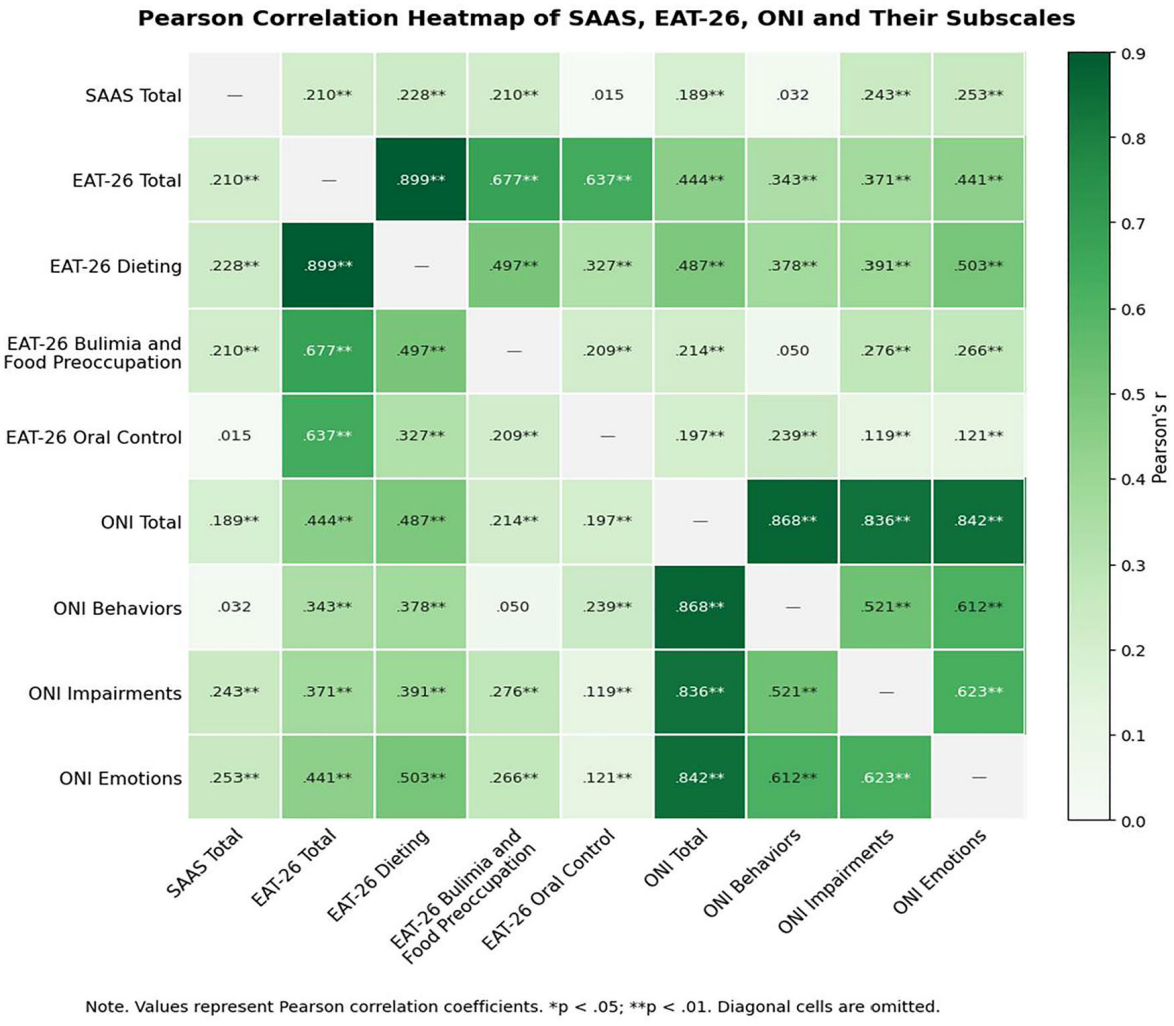
Heatmap illustrating the correlation matrix between SAAS with eating attitudes (also with subscales) and ONI (also with subscales).

The correlation matrix among SAAS total with EAT‐26 total, EAT‐26 subscales (dieting, bulimia and food preoccupation, and oral control), ONI total, and ONI subscales (behaviors, impairments, and emotions) is illustrated in Figure 1. The SAAS total score demonstrated positive correlations with both EAT‐26 (total and also dieting, bulimia and food preoccupation subscales) and ONI (total and also impairments and emotions subscales).

## 4 Discussion

This study examined the predictive role of social appearance anxiety for disordered eating attitudes and ON in a large adult sample. The findings revealed that social appearance anxiety was positively associated with both disordered eating attitudes and ON and remained a significant associated factor even after adjusting for potential confounding variables. These results suggest that social appearance anxiety may be one of the key psychological factors underlying maladaptive eating behaviors. Also, the present study also examined the associations between social appearance anxiety and participants' sociodemographic and lifestyle characteristics. Women reported significantly higher social appearance anxiety than men, consistent with previous studies (Hagger et al. 2010; Hawes et al. 2020). This finding may be explained by stronger sociocultural pressures regarding body image, thinness ideals, and appearance‐based evaluation among women (Aparicio‐Martinez et al. 2019). In addition, single participants exhibited higher social appearance anxiety than married individuals. Although Sanlier et al. (2018) found no association with marital status, our findings may reflect greater appearance‐related concerns during periods of active social interaction and partner selection. Consistent with Klos and Sobal (2013), interpersonal relationships may play an important role in shaping appearance‐related concerns.

Furthermore, participants with lower educational attainment and lower physical activity levels had significantly higher social appearance anxiety. While previous findings regarding educational level have been inconsistent (Hawes et al. 2020), lower educational attainment may be associated with greater vulnerability to appearance‐related pressures. Similarly, regular physical activity has been linked to more positive body image and lower appearance‐related anxiety, which may explain the lower SAAS scores observed among physically active participants. Overall, these findings suggest that social appearance anxiety is influenced not only by psychological factors but also by broader sociodemographic and lifestyle characteristics.

One of the key findings of this study is the positive association between social appearance anxiety and disordered eating attitudes. This finding is consistent with previous research demonstrating a strong relationship between social anxiety‐related constructs and disordered eating behaviors. For instance, it has been reported that social anxiety symptoms are closely linked with various forms of disordered eating, including dietary restraint, binge eating, and compensatory behaviors (Linardon et al. 2017). Furthermore, social appearance anxiety has been reported to be a significant associated factor of eating disorder symptoms and to strengthen the relationship between internalization of the thin body ideal and eating disorder symptoms (Christian et al. 2021). In addition, social appearance anxiety has been identified as a specific associated factor of eating disorder symptoms, highlighting the importance of appearance‐based concerns in the etiology of maladaptive eating behaviors. The present findings contribute to the literature by demonstrating that social appearance anxiety is associated not only with clinical eating disorders but also with disordered eating patterns that fall below the clinical diagnostic threshold.

Another important finding is a significant relationship between social appearance anxiety and orthorexia nervosa. Although ON has traditionally been conceptualized as a health‐driven behavior, emerging evidence suggests that it may share underlying psychological mechanisms with other forms of disordered eating. Previous studies have shown that orthorexia nervosa is associated with disordered eating attitudes and related cognitive and behavioral patterns (Bartel et al. 2020; Hallit et al. 2021). Similarly, meta‐analytic findings indicate a moderate association between ON symptoms and eating disorder symptoms, suggesting partial overlap between these constructs (Zagaria et al. 2022). The current study supports this perspective by demonstrating that individuals with higher social appearance anxiety also exhibit higher ON tendencies. This implies that concerns related to appearance and social evaluation may also play a role in behaviors that are ostensibly motivated by health.

The findings also revealed a positive association between disordered eating attitudes and ON. The literature reports that disordered eating attitudes are associated with ON and that these two conditions frequently coexist (Obeid et al. 2021). For example, it has been shown that higher EAT‐26 scores are significantly associated with increased ON behaviors, even after controlling for additional psychological variables (Obeid et al. 2021). Moreover, studies on young adults have demonstrated that individuals with higher ON levels also report higher disordered eating attitudes (Brytek‐Matera et al. 2022; Kaya et al. 2024b; Kaya et al. 2022). These present findings are consistent with these results and further support the conceptualization of orthorexia nervosa as a construct that is closely related to, yet distinct from, disordered eating patterns. Anorexia nervosa, often compared to orthorexia nervosa among eating disorders, shares similarities in perfectionism and the need for control; however, the most significant difference with orthorexia nervosa is its focus on the quality of food rather than the quantity. Individuals with anorexia nervosa and bulimia nervosa may consume less dense and healthier foods due to fears of weight gain, loss of control, and binge eating, stemming from behaviors involving control over body shape and weight. Individuals with orthorexia nervosa, on the other hand, prioritize food purity and therefore prefer higher‐quality foods to enhance their health benefits (Zagaria et al. 2022). In this respect, their perspectives and consequently their food preferences differ.

The regression analyses have shown that social appearance anxiety is a significant associated factor of both disordered eating attitudes and orthorexia nervosa. Even after adjusting for sociodemographic variables such as age, gender, marital status, and physical activity level, social appearance anxiety remained a significant associated factor of both disordered eating attitudes and ON. This finding suggests that the relationship between social appearance anxiety and maladaptive eating behaviors is robust and not solely explained by demographic or lifestyle factors. Previous studies have also emphasized the importance of underlying psychological vulnerabilities in explaining the co‐occurrence of anxiety and disordered eating patterns (Levinson and Rodebaugh 2016). In this context, social appearance anxiety may represent a shared vulnerability that contributes to both disordered eating attitudes and orthorexic tendencies. Perfectionism has been shown to be linked to behavioral problems, including eating disorders, anxiety, and anxiety‐related disorders (Lunn et al. 2023). A study conducted on young women has shown that young women with high levels of perfectionism are also at higher risk of eating disorders (Mandiola et al. 2022). Perfectionist individuals may experience social anxiety because this behavior leads them to be more sensitive about their physical appearance and to care about the opinions of others (Aygün and Akbağ 2025). It emphasizes that perfectionism is associated with both social anxiety and social apprehension because it shapes expectations in social relationships (Wang et al. 2022). A study using Cognitive Behavioral Therapy‐based group sessions for young adults to develop coping strategies for social anxiety, focusing on improving self‐esteem and body image, showed that improvement in social anxiety can lead to positive changes in self‐esteem and body image (Akkaya and Artan 2025). Another factor associated with social anxiety is social media use (Wu et al. 2024). These results highlight the importance of interventions including Cognitive Behavioral Therapy and media literacy training, as well as self‐awareness, in preventing and treating social appearance anxiety.

This study has several limitations. First, the cross‐sectional design does not allow for the establishment of causal relationships. In addition, the use of self‐reported data may introduce the risk of measurement bias. Furthermore, although the Orthorexia Nervosa Inventory (ONI) is a validated multidimensional instrument, it assesses orthorexic tendencies rather than providing a clinical diagnosis, and orthorexia nervosa itself currently lacks universally accepted diagnostic criteria and a validated clinical cut‐off score. Therefore, the findings related to ON should be interpreted with caution. We were unable to report ON prevalence because there is no validated cut‐off point for the ONI in the Turkish population. Future research should employ longitudinal and experimental designs to further elucidate the causal role of social appearance anxiety in eating behaviors. Moreover, investigating the underlying psychological mechanisms linking ON and disordered eating attitudes—such as perfectionism, emotion regulation difficulties, and body dissatisfaction—would contribute significantly to the literature. Studies in different cultural samples are important in revealing the generalizability of these relationships.

## 5 Conclusion

In conclusion, social appearance anxiety appears to be an important psychological factor associated with both disordered eating attitudes and orthorexia nervosa. Furthermore, social appearance anxiety was significantly associated with gender, marital status, educational level, and physical activity, suggesting that both psychological and sociodemographic factors should be considered when evaluating maladaptive eating behaviors. These findings emphasize the importance of adopting a holistic approach that integrates psychological and nutritional perspectives. From a practical perspective, assessing appearance‐related concerns during nutritional assessment may help dietitians identify individuals at greater risk for disordered eating attitudes and orthorexic tendencies, thereby supporting more individualized and effective nutrition counseling. In addition, interventions targeting social appearance anxiety, such as cognitive behavioral approaches, media literacy programs, and mindfulness‐based strategies, may represent promising avenues for the prevention and management of maladaptive eating behaviors. Future longitudinal and intervention studies are warranted to clarify the causal relationships among these constructs and to evaluate the effectiveness of such approaches.

## Author Contributions

**Funda Pınar Çakiroğlu**: conceptualization, methodology, validation, Writing – original draft, Writing – review and editing, supervision. **Gökmen Zararsiz**: conceptualization, formal analysis, validation, writing – review and editing. **Zeynep Uzdil**: conceptualization, methodology, investigation, writing – original draft, writing – review and editing, formal analysis, validation. **Seda Kaya**: conceptualization, methodology, investigation, validation, formal analysis, writing – original draft, writing – review and editing.

## Funding

The authors have nothing to report.

## Ethics Statement

This study was conducted according to the guidelines laid down in the Declaration of Helsinki and all procedures involving research study participants were approved by the Clinical Research Ethics Committee of Ondokuz Mayıs University.

## Consent

Online informed consent was obtained from all subjects.

## Conflicts of Interest

The authors declare no conflicts of interest.

## Data Availability

The datasets used and analyzed during the current study are available from the corresponding author upon reasonable request.
